# Incidence, circulation, and spatiotemporal analysis of seasonal influenza in Shandong, China, 2008–2019: A retrospective study

**DOI:** 10.1111/irv.12959

**Published:** 2022-01-11

**Authors:** Ti Liu, Ping Wang, Fanyu Meng, Guoyong Ding, Julong Wu, Shaoxia Song, Lin Sun, Shengyang Zhang, Zhong Li, Weijia Xing, Xianjun Wang

**Affiliations:** ^1^ Shandong Provincial Key Laboratory of Infectious Disease Control and Prevention Shandong Center for Disease Control and Prevention Jinan China; ^2^ School of Public Health Shandong First Medical University and Shandong Academy of Medical Sciences Tai'an China; ^3^ Statistical Analysis Center Linyi Central Hospital Linyi China

**Keywords:** incidence, predominant strains, seasonal influenza, spatiotemporal pattern

## Abstract

**Background:**

Understanding the influenza‐like illness (ILI) incidence, circulation pattern of virus strains and spatiotemporal pattern of influenza transmission are important for designing control interventions. Based on the 10 years' surveillance data, we aimed to provide a baseline characterization and the epidemiology and dynamics of influenza virus in Shandong.

**Methods:**

We extracted surveillance and laboratory testing data. We estimated the ILI incidence and analyzed the predominant virus. A wavelet power analysis was used to illustrate the periodicity. In addition, we applied a linear regression model to characterize the correlation of influenza seasonality with longitude.

**Results:**

The average ILI incidence was estimated to be 3744.79 per 1 million (95% confidence interval [CI]: 2558.09–4931.45) during 2009–2018. Influenza A/H1N1 and A/H3N2 strains predominated in the most influenza seasons in Shandong. The annual amplitude of influenza epidemics decreased with longitude (*P* < 0.05). In contrast, the epidemic peak of influenza emerged earlier in the western region and increased with longitude in influenza A (*P* < 0.05). The annual peak of the influenza B epidemic lagged a median of 4.2 weeks compared with that of influenza A.

**Conclusions:**

The development or modification of seasonal influenza vaccination strategies requires the recognition that the incidence is higher in preschool‐ and school‐aged children. Although seasonal influenza circulates annually in Shandong, the predominant virus strain circulation pattern is extremely unpredictable and strengthening surveillance for the predominant virus strain is necessary. Lower longitude inland regions need to take nonpharmaceutical or pharmaceutical interventions in advance during influenza high‐occurrence seasons.

## 1 INTRODUCTION

Influenza A virus and influenza B virus are two common types of influenza viruses that cause human respiratory disease. The outbreak of seasonal influenza usually occurs in autumn and winter in the Northern Hemisphere and leads to a huge disease burden, particularly in children.^1^ Despite decades of surveillance and influenza virus vaccine interventions, seasonal influenza viruses continue to cause epidemics around the world each year. From 2010 to 2015 in China, more than 80,000 influenza‐associated excess respiratory deaths were reported every year.^2^ Most notably, the reported number of influenza cases in the 2017–2018 influenza season ranked second in China after the 2009 pandemic H1N1.^3^, ^4^

Shandong province is a coastal region in eastern China and faces the Korean Peninsula and Japanese Islands. It belongs to warm temperate zone with semitropical monsoon climate types.^5^ Shandong as one of the highest population densities among provinces in China,^6^ the threat of seasonal influenza outbreaks should not be underestimated. It is necessary to systematically analyze the geographical variations in the patterns of seasonal influenza and the dynamics of the predominant virus in Shandong to improve outbreak prevention strategies. Therefore, we aimed to provide a baseline characterization and the epidemiology and dynamics of influenza virus by evaluating influenza‐like illness (ILI) incidence rates, the shift of predominant virus, positive rates, and seasonal patterns of influenza A and B over a 10‐year period (i.e., 2009–2018) in Shandong.

## 2 MATERIALS AND METHODS

### 2.1 Case definitions

The national ILI surveillance program was initiated by the Ministry of Health in 2000 consistent with the recommendations by the World Health Organization (WHO).^7^ Influenza surveillance and technical guidance were updated in 2017 and outlined by the National Health and Family Planning Commission of the People's Republic of China.^8^, ^9^ According to the guidance, the ILI case was defined as an individual with fever (≥38°C) and other nonspecific symptoms, including cough, and sore or dry throat. Influenza‐confirmed cases require isolation of influenza virus by culture or detection of influenza virus by nucleic acid testing from a nasopharyngeal swab. The confirmed influenza cases were classified as for influenza A and B infection.

### 2.2 Specimens collection and laboratory testing

All sentinel hospitals collected the required number of clinical specimens every week or every month. Sentinel hospitals in Shandong were required to collect 10–15 nasopharyngeal swabs every week from October to March of the next year and 10–15 nasopharyngeal swabs every month from April to September.^8^

The collected specimens were immediately placed in viral transport medium (VTM) and stored at 2–8°C at local hospitals. These specimens were transferred to the closest influenza network laboratory within 48 h of collection to identify the type/subtype of influenza virus by real‐time reverse transcription polymerase chain reaction (PCR) or hemagglutination inhibition after virus isolation. If the specimens could not be sent to the laboratory within 48 h of collection, they were stored at <70°C and sent to the laboratory within 1 week. These specimens were used to inoculate Madin‐Darby canine kidney (MDCK) cells. Viral RNA was extracted from specimens and viruses using the QIAamp Viral RNA Minikit (QIAGEN, Hilden, Germany, cat. 52904) according to the manufacturer's instructions. The primer and probe sequences were provided by the China National Influenza Center (CNIC) to all influenza surveillance network laboratories according to the WHO Information for the Molecular Detection of Influenza Viruses.^10^ Specimens were first classified as influenza A or B infection. Most influenza‐positive samples were further tested to identify different subtypes of influenza virus. Influenza A included A/H1N1, B/H3N2, and A/unsubtyped. Influenza B consisted of B/Victoria, B/Yamagata, and B/unsubtyped. All testing was performed in biosafety level two facilities. Influenza virus strains were defined as predominant circulating viruses if they made up the largest proportion of positive samples by type or subtype during an influenza season.^11^ Any specimens that were not identified as serotypes by RT–PCR or cytology were sent to the national influenza center for review.

### 2.3 Data analysis

All reported ILI cases from April 1, 2009, to March 30, 2019, by sentinel hospitals in Shandong were included in our analysis, which covered 10 surveillance years. A surveillance year was defined as a year from the 14th week of the year to the 13th week of the next year. The epidemiological patterns of ILI cases and influenza A and B cases were studied. The age‐specific and yearly infection rates of influenza were estimated. We also categorized the 17 cities into two zones (inland zone and coastal zone) on the basis of whether the city had a coastline. The coastal zones contain 7 coastal cities, and the inland zones contain 10 inland cities (Figure S1).

A previous study used a multiplier model approach to estimate the ILI incidence rate.^12^ We also used the same models to estimate the incidence rate of ILI. We first estimated the number of ILIs and then divided by the population size from the Shandong Provincial Bureau of Statistics. The total number of ILIs and the equation are described in

$$T_{\mathrm{ili}}=\sum \frac{N_{a}\times R_{a}}{P_{s}\times Q\times S\times T},$$

where $T_{\mathrm{ili}}$ are the year number of influenza‐associated outpatient visits, the $N_{a}$ was an age‐specific reported number of ILI consultations from ILI surveillance, the $R_{a}$ was the age‐specific proportion of positive cases from influenza virus surveillance, and the $P_{s}$ was the proportion of symptomatic cases among influenza infections. The parameter $P_{s}$ was obtained from a review study and ranged from 58.3% to 74.5%,^13^ the Q was the proportion of ILI cases among symptomatic infections,^12^ the S was the success rate for sampling pharyngeal swab specimens, which ranged from 80% to 90%,^12^ and the T was the test sensitivity of detection of PCR, which ranged from 95% to 100%.^12^ We used Monte Carlo simulation to estimate the incidence rate 95% confidence intervals (CIs) by the mean ± 1.96 times the standard deviation.

To analyze the periodicity of influenza cases in Shandong, we conducted wavelet analysis based on the weekly number of influenza A and B infected cases using the Morlet function.^14^ In addition, we further studied the seasonal parameters following Fourier analysis.^15^ The linear regression model estimates the peak timing and amplitude of the annual and semiannual periodicities of influenza activity in each city, as described in

$${\mathrm{flu}}_{i}\left(t\right)=a_{i}+b_{i}\times \cos\left(2\pi \times t/52.17\right)+c_{i}\times \sin\left(2\pi \times t/52.17\right)+d_{i}\times \cos\left(4\pi \times t/52.17\right)+e_{i}\times \cos\left(4\pi \times t/52.17\right)$$

where ${\mathrm{flu}}_{i}\left(t\right)$ are the weekly standardized number of influenza‐positive A or B in cities i; t is a running index for week; and $a_{i},b_{i},c_{i},d_{i}$, and $e_{i}$ are the intercept and seasonality coefficients to be estimated from the data.

Specifically, the amplitude of the annual periodicity is estimated as ${\text{AnnAmp}}_{i}=\text{sqrt}\left(b_{i}^{2}+c_{i}^{2}\right)$, the annual peak timing is estimated as ${\text{AnnPeakTiming}}_{i}=-\text{atan}\left(c_{i}/b_{i}\right)$, the amplitude of the semiannual periodicity is estimated as ${\text{AnnPeakTiming}}_{i}=-\text{atan}\left(c_{i}/b_{i}\right)$, and the semiannual peak timing is estimated as ${\text{SemiAnnAmp}}_{i}=\text{sqrt}\left(d_{i}^{2}+e_{i}^{2}\right)$.

To predict the effects of influenza seasonality in Shandong geographic regions, we fitted seasonal regression models in all 17 cities using a dependence variable for longitude. In seasonal regression models weighted by the weekly number of ILI cases, we were able to reduce the potential for systematic differences to be caused by the city's population. The Shandong map was based on the public geographical data downloaded from the Resource and Environment Science and Data Center, Institute of Geographic Sciences and Natural Resources Research, CAS (https://www.resdc.cn/).

All analyses and illustrations were performed with R software (Version 4.1.0, R Foundation for Statistical Computing). Two‐sided *P* values of <0.05 were considered to indicate statistical significance.

## 3 RESULTS

### 3.1 Characteristics of ILI cases

From 2009 to 2018, 2,548,416 ILI cases from the sentinel hospitals of Shandong were reported to the surveillance system. The average incidence rate of ILI was 3744.77 per 1 million persons during 2009–2018 (Table 1). The incidence rate of ILI was high (4845.39/million persons) due to the A/H1N1 pandemic in 2009. Incidence rates decreased in the following years, until 2015, when the incidence rates rebounced and reached a peak in 2017 (11,841.97/million persons; Table 1). The incidence rates of ILI were inversely associated with age (Table 2). The incidence rate was highest in the cases aged under 4 years (23,116.10/million persons) and lowest in the elderly individuals aged over 60 years (632.05/million persons).

**TABLE 1:** Estimated incidence rates of influenza‐like illness by surveillance year

Surveillance years	Incidence rates (per 1 million persons)	95% CI incidence rates (per 1 million persons)
2009	4845.39	3309.93–6380.85
2010	785.35	536.48–1034.23
2011	1360.75	929.54–1791.96
2012	1156.68	790.14–1523.22
2013	1352.96	924.22–1781.70
2014	1761.90	1203.57–2320.22
2015	3023.50	2065.38–3981.62
2016	3507.09	2395.73–4618.45
2017	11,841.97	8089.37–15,594.58
2018	7419.35	5068.23–9770.47
Average	3744.77	2558.09–4931.45

Abbreviation: CI, confidence interval.

**TABLE 2:** Estimated incidence rates of influenza‐like illness by age group

Age groups (years)	Incidence rates (per 1 million persons)	95% CI incidence rates (per 1 million persons)
0–4	23,116.10	15,790.84–30,441.37
5–14	13,399.98	9153.66–17,646.30
15–24	2249.14	1536.41–2961.88
25–59	933.59	637.74–1229.43
≥60	632.05	431.76–832.34

Abbreviation: CI, confidence interval.

### 3.2 Characteristics of influenza A and B cases

From 2009 to 2018 surveillance years, 169,362 patients were tested for influenza virus infection. Of whom 28,729 (16.96%) patients tested positive. Of these, 19,420 (67.60%) patients were confirmed to have influenza A infection, and the remaining 9309 (32.40%) patients were confirmed to have influenza B infection. The median age of influenza A cases was 11 years (interquartile range [IQR]: 4–26 years), and of influenza B cases was 13 years (IQR: 5–15 years). The male to female ratio of laboratory‐confirmed cases was 1.16:1, and the sex distribution in influenza A and B cases was similar (*P* > 0.05).

**FIGURE 1:**
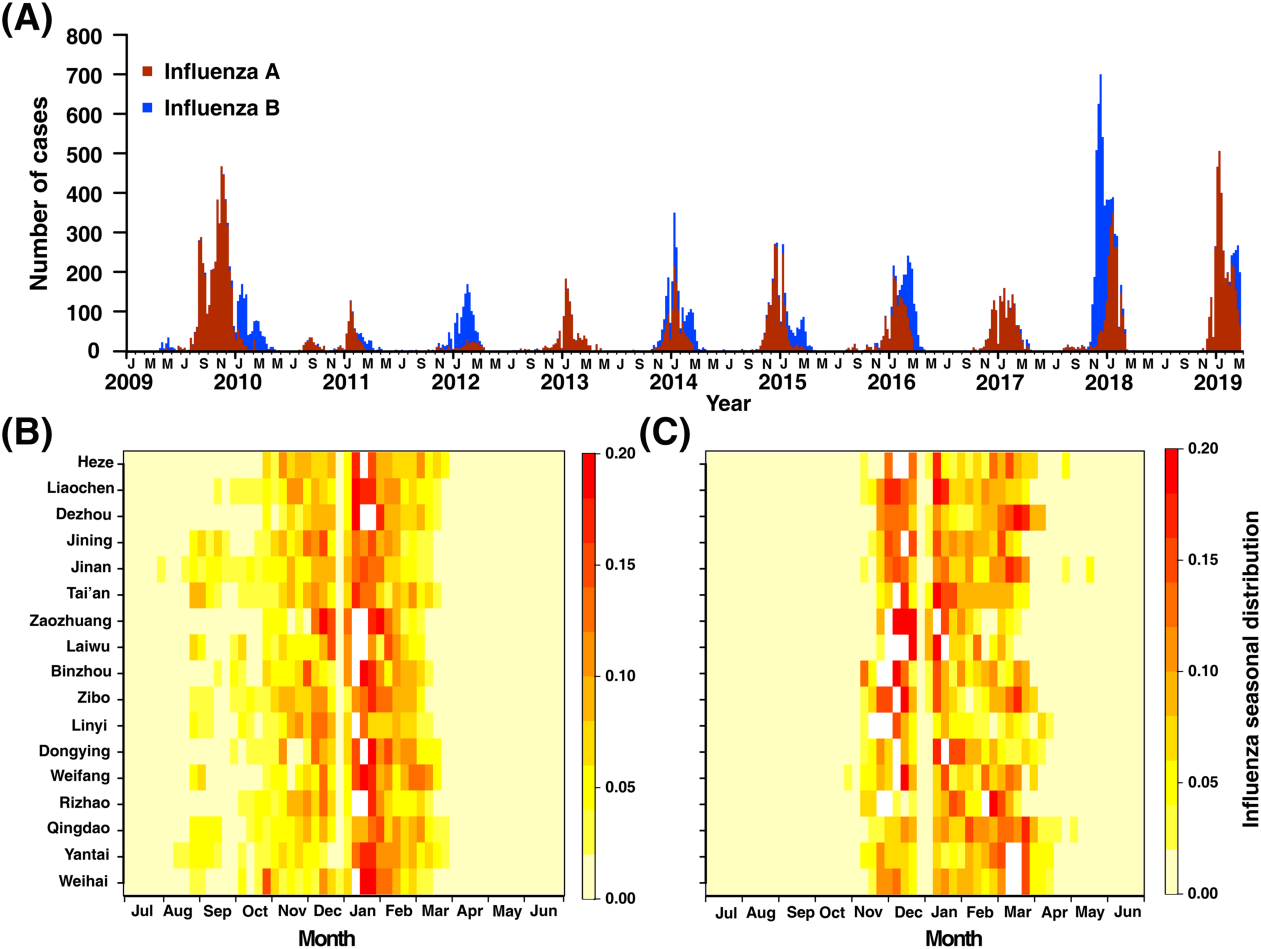
Heatmap of surveillance data for influenza A and B by cites of Shandong, 2009–2018. (Panel A) Weekly number of laboratory‐confirmed cases of influenza A and B. (Panel B) Seasonal distribution of influenza A cases, plotted as the median value of proportion of cases in each week of the year from 2009 to 2018. (Panel C) Seasonal distribution of influenza B cases, plotted as the median value of proportion of cases in each week of the year from 2009 to 2018. For Panel (A) and Panel (B), the cities were ordered by increasing longitude from western (top) to eastern (bottom)

Except for the 2009 surveillance year in which the outbreak was strongly affected by the 2009 A/H1N1 pandemic, the seasonal influenza epidemic usually started in November and ended in March or April of the next year. A single annual peak of influenza was observed in Shandong province (Figure 1A). A lag of 1 month was observed between the peaks of influenza A and B outbreaks. The number of influenza A cases increased from August and reached a peak in January (Figure 1B). The number of influenza B cases increased after November. Nevertheless, the peak time of the influenza B outbreak varied with longitude. A lag of 2 months was observed between the inland cities and the coastal cities (Figure 1C). Moreover, the peak influenza B outbreak arrived much earlier in the 2018 surveillance year, in which an intense increase in the number of influenza B cases was observed (Figure 1A).

**FIGURE 2:**
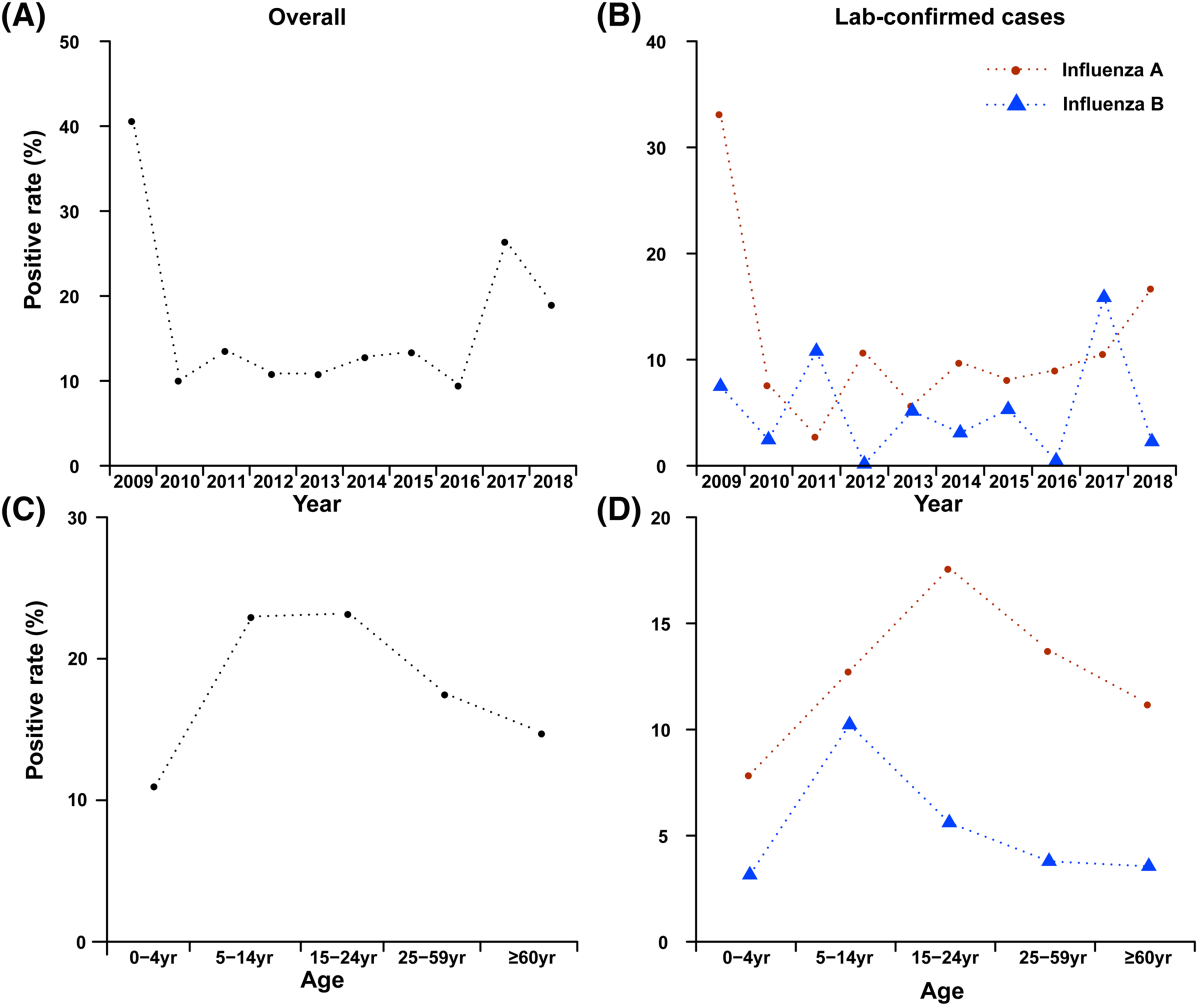
Age and time distribution of infection rates of influenza A and B

The average infection rate of influenza virus was 17.06% during 2009–2018. The majority of cases were infected with influenza A, and the positive rate of influenza A infection was 11.53%. The positive rate of influenza B was 5.53%. The infection rates of influenza virus varied largely with time in Shandong. The highest infection rate was observed in the 2009 surveillance year (40.67%), and the lowest was observed in 2016 (9.50%). The infection rates in other years ranged from 10.09% to 26.44% (Figure 2A). The infection rate of influenza A was also highest in the 2009 surveillance year due to the A/H1N1 pandemic. In the remaining years, the infection rates of influenza A ranged from 2.79% to 16.75% (Figure 2B). However, the infection rates of influenza B presented a biennial peak (Figure 2B). The influenza‐positive rates varied by age group (*P* < 0.001). The overall influenza‐positive rate was highest among those aged 15–24 years (23.21%, 4187/18,043), followed by 5–14 years (22.98%, 10,807/47,018), 25–59 years (17.52%, 5731/32,716), >60 years (14.76%, 889/6023), and 0–4 years (11.02%, 7115/64,562; Figure 2C). The positive rate of influenza A was higher than that of influenza B in all age groups and was highest among those aged 15–24 years (17.60%), but the positive rate of influenza B was highest among those aged 5–14 years (10.23%; Figure 2D).

**FIGURE 3:**
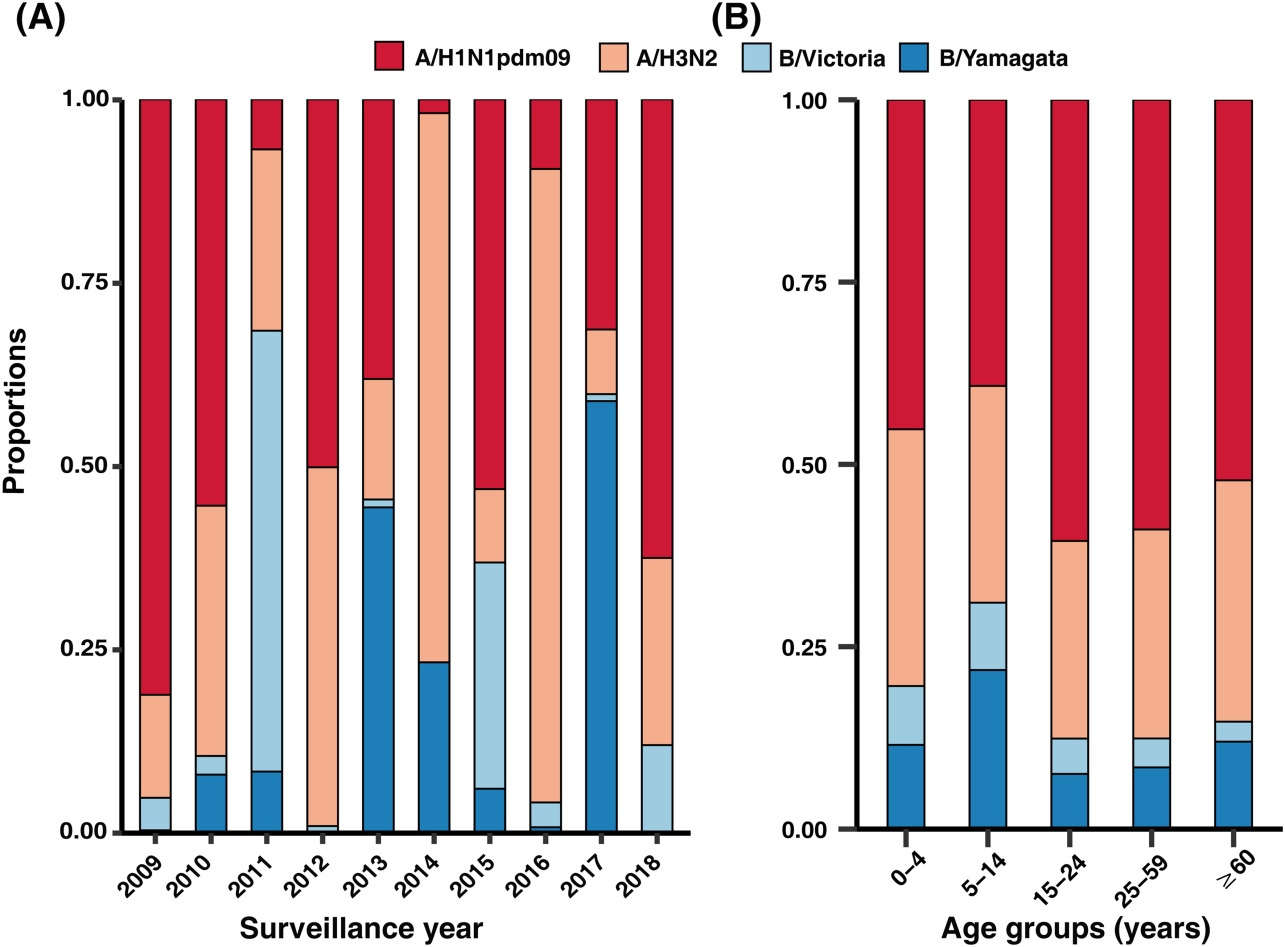
Proportions of influenza pathogens in laboratory‐confirmed cases in Shandong. (Panel A) Proportions of influenza pathogens by year. (Panel B) Proportions of influenza pathogens by age group

The circulating pathogens of influenza virus shifted frequently in Shandong. Because of the 2009 A/H1N1 pandemic, the proportions of A/H1N1 virus were dominant in 2009 and 2010 (81.22% and 55.42%, respectively; Figure 3A). In 2011, B/Victoria replaced A/H1N1 and became the predominant pathogen (60.12%). However, most influenza cases were caused by A/H1N1 infection in 2012 (50.18%). After that, A/H1N1 circulated again in 2015 (53.16%), 2017 (31.41%), and 2018 (62.55%). Moreover, A/H3N2 was the other circulating pathogen in Shandong. Indeed, A/H3N2 became the predominant pathogen in 2014 and 2016 (74.90% and 86.40%, respectively). In the remaining years, although influenza B was at a low activity level in 2009, 2010, 2012, 2016, and 2018, B/Victoria and Yamagata became the predominant pathogen in 2011 (44.34%) and 2017 (58.79%), respectively (Figure 3A). The pathogen proportion was significantly different in different age groups (*P* < 0.001). A/H1N1 was the predominant pathogen in all age groups (range: 33.42%–55.76%; Figure 3B). H3N2 was the other important influenza virus that infected many cases. Although influenza A predominated in the majority of the seasons considered, influenza B infection also played a significant role in seasonal epidemics.

### 3.3 Local influenza dynamics

**FIGURE 4:**
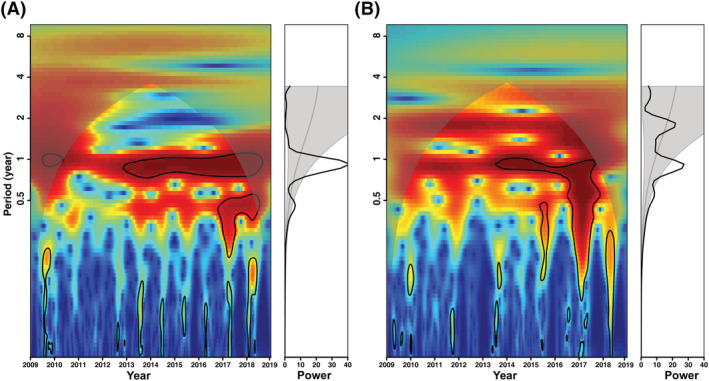
Local wavelet power spectrum for influenza A and B epidemics. Every panel on the left shows the wavelet power spectrum where (i) the value increases from blue to red, cold color‐related low‐power regions (green to blue), whereas warm areas (yellow to red) show high power. (ii) Black contour lines indicate the 95% confidence interval; (iii) shaded regions on both ends delineate the cone of influence, estimates of which may be affected by the edge effect and therefore should be interpreted with caution. On the right, the graph represents the average wavelet power over time, where the shaded regions indicate significant periods. The black line beyond the regions indicates that the period was statistically significant

**FIGURE 5:**
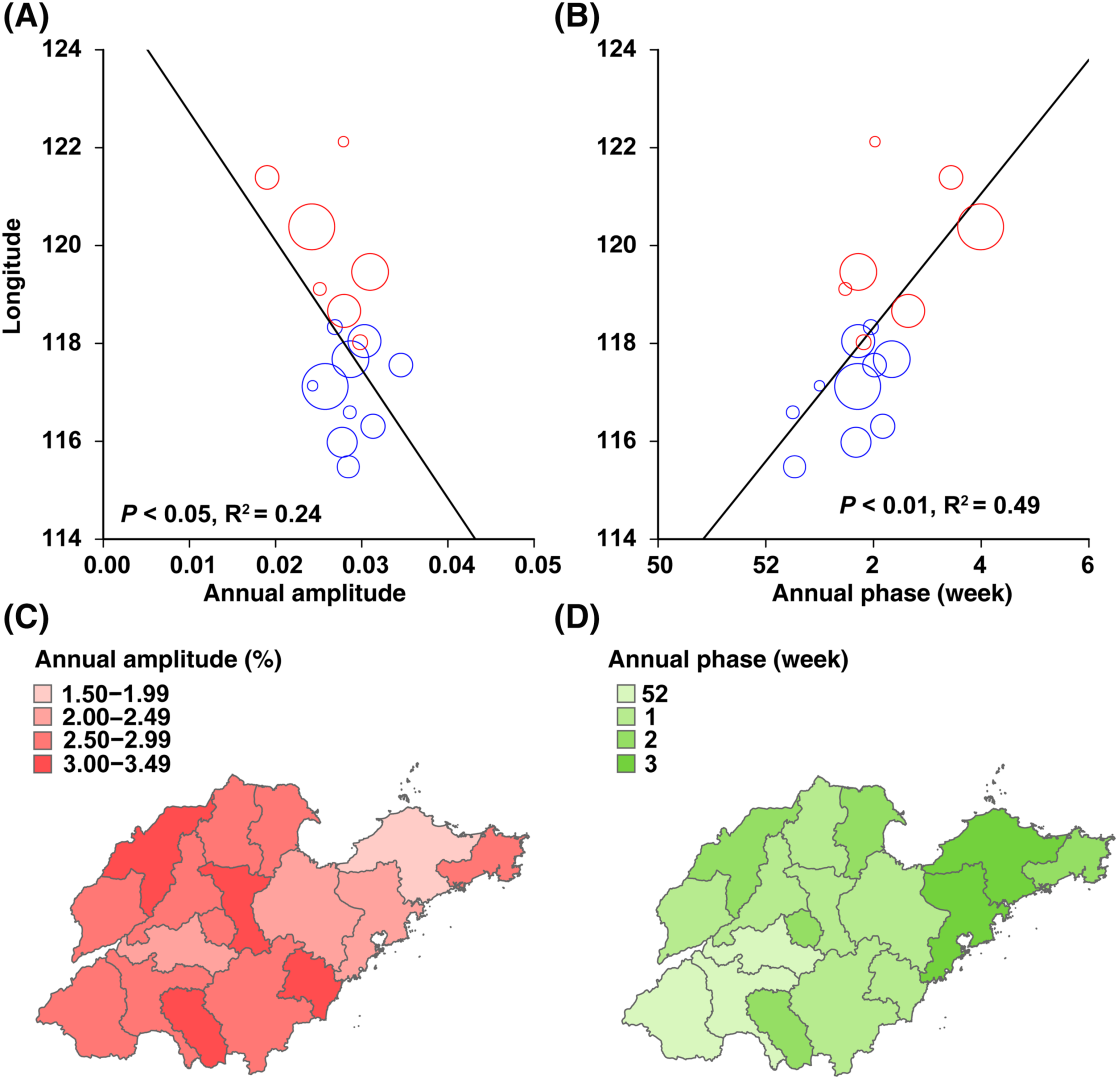
Longitude gradients in periodicity and peak time of the influenza A epidemic. (Panel A) Amplitude of the annual periodicity. (Panel B) Peaking time of the annual periodicity. Symbol size is proportional to the number of influenza‐like illness (ILI) cases in each city. Black solid lines represent linear regression fit (regression weighted by mean annual number of cases of ILI cases). *P* and *R* ^2^ values are given on the graphs. Colors represent different city types (red = coastal zone, blue = inland zone). (Panel C) Amplitude of the annual cycle from pale red (low) to red (high). (Panel D) Peaking time of the influenza B epidemic, in weeks from Jan 1st. Timing is color coded from pale green (low) to green (high)

**FIGURE 6:**
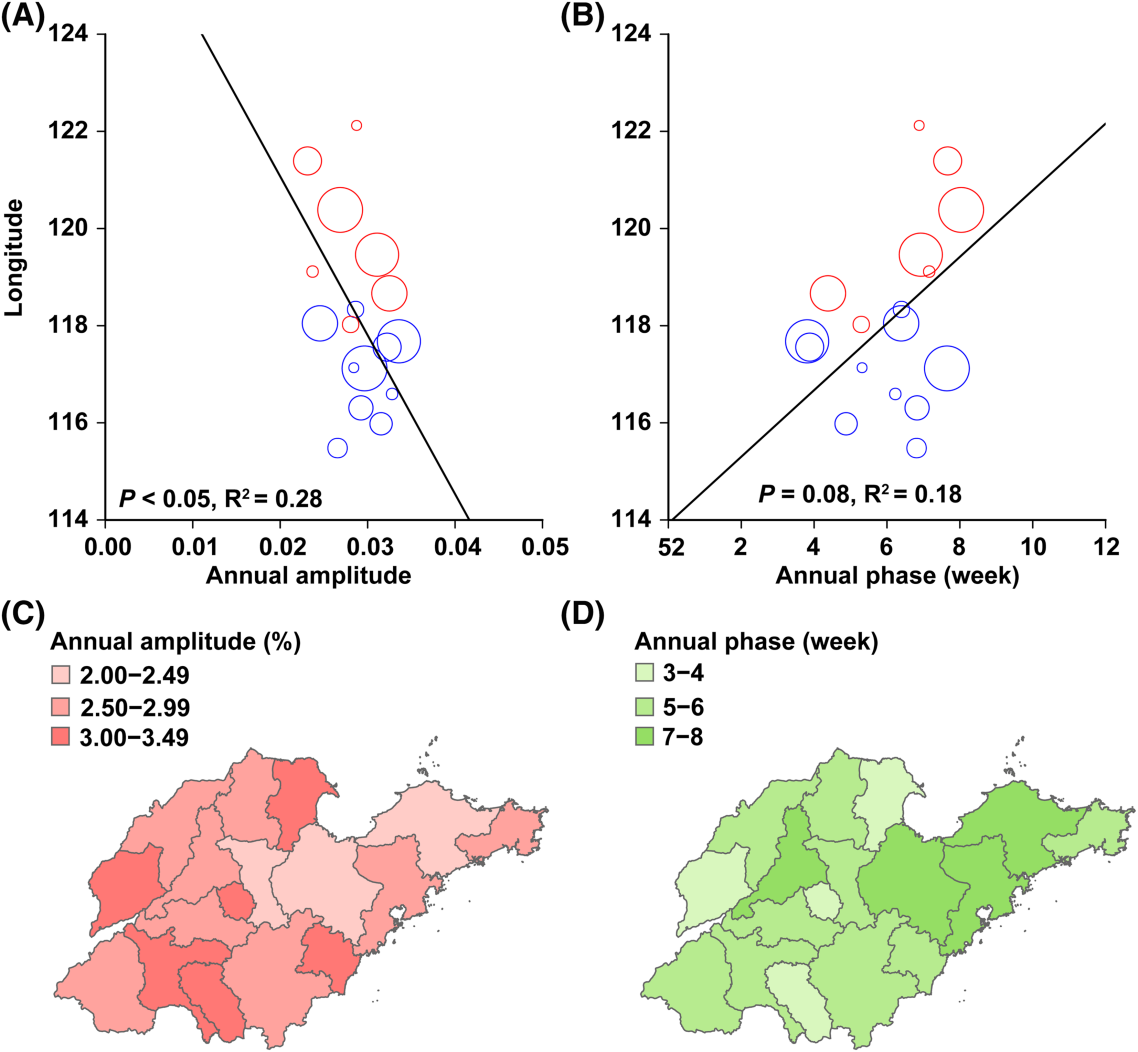
Longitude gradients in periodicity and peak time of the influenza B epidemic. (Panel A) Amplitude of the annual periodicity. (Panel B) Peaking time of the annual periodicity. Symbol size is proportional to the number of influenza‐like illness (ILI) cases in each city. Black solid lines represent linear regression fit (regression weighted by mean annual number of cases of ILI cases). *P* and *R* ^2^ values are given on the graphs. Colors represent different city types (red = coastal zone, blue = inland zone). (Panel C) Amplitude of the annual cycle from pale red (low) to red (high). (Panel D) Peaking time of the influenza B epidemic, in weeks from Jan 1st. Timing is color coded from pale green (low) to green (high)

The results of wavelet analysis showed that the largest powers were at the period of 1 year, and a significant annual periodicity was observed for influenza A and B epidemics (Figure 4A,B). The annual amplitude of influenza A and B epidemics increased with decreasing longitude, and annual amplitude periodicity was strongest in the West (Figures 5A,C and 6A,C). The annual peak timing of influenza A activity occurred earlier than that of influenza B. The annual peak timing of the influenza A epidemic ranged from the 52nd to 3rd week of the next year, and the influenza B epidemic reached a peak at approximately the 3rd to 8th weeks (Figures 5B,D and 6B,D). The peak of influenza A and B epidemics arrived earlier in inland cities than in coastal cities. In addition, a semiannual periodicity was identified for the influenza B epidemic since the 2015 surveillance years (Figure 4B). Contrary to the annual amplitude, the semiannual amplitude of the influenza B was not related to longitude (Figure S2A,C). However, the semiannual peak time was also significantly related to longitude. The influenza B epidemic in inland cities reached a peak earlier than that in coastal cities (Figure S2B,D).

## 4 DISCUSSION

To our knowledge, this is the first comprehensive study of ILI burden and epidemiological characteristics of influenza in Shandong. Our study revealed that generally influenza A is relatively more active than influenza B, and seasonal epidemics occur between August and April of the next year. We estimated that the average incidence rate of ILI was 3744.77 per 1 million persons during 2009–2018, mainly in preschool‐ and school‐aged children. Although influenza A predominated in the majority of seasons, influenza B also played a significant epidemic role in the population. In particular, B/Yamagata became a predominant virus and caused a large epidemic in Shandong during 2017–2018. The influenza A and B epidemics occurred annually in Shandong, while a semiannual and weaker 2‐year periodicity of the influenza B epidemic was also revealed by our results. We reported evidence of a West‐to‐East spatiotemporal pattern of spread of influenza A and B across Shandong.

The ILI incidence rates in Shandong during 2009–2018 ranged from 785.35 to 11,841.97 per million. Globally, the ILI incidence rate was much lower than that of Guangdong and Beijing.^16^, ^17^ Our age profile of the ILI incidence rate is in agreement with reports from other studies; the incidence rate peaked at <5 years of age and declined with increasing age.^12^, ^18^ Findings from Beijing also showed that preschool‐ and school‐aged children had the highest incidences of morbidity due to seasonal influenza.^12^ Therefore, seasonal influenza vaccination strategies should be considered to target preschool‐ and school‐aged children. Regrettably, in China, influenza vaccination is still mostly self‐paid and not included in the National Immunization Program. Influenza vaccination is rarely included in the government finance‐reimbursed policy in China.^19^ Beijing, the capital of China, has invested approximately 30.5 million RMB ($US 5.1 million) each year to provide free influenza vaccination to seniors (≥60 years) and all primary and secondary school students since 2007.^20^ However, in Shandong, only a small portion of counties implementing influenza vaccines include the New Rural Cooperative Medical Insurance for Rural Residents for all ages or those aged over 60 years.^19^

Our data suggest that influenza A and B epidemics arise annually in Shandong, in contrast with YiChang in southern China, where seasonal influenza circulates in annual, semiannual, and year‐round epidemics. Similarly, annual epidemics were reported in Beijing, Liaoning province, which is also located in northern China.^21^ In addition, although seasonal influenza circulates annually in Shandong, the predominant virus strain circulation pattern is extremely complicated. Indeed, a change in the relative predominance of the B/Yamagata strains was observed during the 2017–2018 influenza high‐occurrence seasons, which contributed to a large influenza epidemic and posed great challenges to public health. We postulate that at least two factors play keyed roles in this phenomenon. First, the trivalent influenza vaccine strain and influenza B epidemic strains were mismatched during the 2017–2018 seasons. This may have affected influenza vaccine effectiveness. Second, B/Yamagata was not the predominant virus strain before 2017, which may have led to a decrease in prior immunity and increased susceptibility.

For seasonal patterns of influenza in Shandong, we demonstrated that longitude rank is a significant predictor. The annual amplitude of influenza A and B epidemics increased with decreasing longitude, and the peak time occurred early in the western regions. We also found evidence of a West‐to‐East spatiotemporal epidemic peak of spread of seasonal influenza A and B across Canada, America, and Europe.^22^, ^23^, ^24^ The special spread pattern was influenced by many factors. In one study, analysis of the spatiotemporal spread of influenza in the United States found a consistent early onset of the epidemic in California, which is the most populous state in the United States.^23^ Similarly, the western regions of inland cities in Shandong are the most populated part and that may also contribute to spatiotemporal transmission. In addition, the spread may be related to climate factors, such as mountain ranges, plains, lakes, and predominant wind direction, which may drive early epidemic activity.^24^ However, the complex spatiotemporal spread of influenza activity is not clearly understood.

Our study has some limitations. First, we were not able to fully assess the disease burden due to lack of mortality and influenza‐associated hospitalization data. Second, for any common, self‐limiting illness, surveillance is not complete, and most cases go undetected because the disorder is asymptomatic, and the patient does not seek formal care or is not diagnosed and reported. Third, our study data were from a passive surveillance system of influenza, and the patients who did not visit the hospitals could not be captured.

## 5 CONCLUSIONS

We sufficiently documented the seasonal influenza burden and the predominant virus and spatiotemporal spread of influenza transmission. We attempted to illustrate both the detection of influenza epidemic trends and the explanation of their interaction with spatiotemporal variations. We anticipate that other epidemic studies based on the combination of genetic and climate data will be a more powerful way to elucidate intriguing spatiotemporal movements in the future.

## AUTHOR CONTRIBUTIONS

**Ti Liu:** Methodology; visualization; writing‐original draft. **Ping Wang:** Methodology; software; visualization; writing‐original draft. **Fanyu Meng:** Methodology; visualization. **Guoyong Ding:** Methodology; visualization. **Julong Wu:** Data curation. **Shaoxia Song:** Resources. **Lin Sun:** Data curation. **Shengyang Zhang:** Data curation. **Zhong Li:** Resources. **Weijia Xing:** Conceptualization; supervision. **Xianjun Wang:** Conceptualization; supervision.

### PEER REVIEW

The peer review history for this article is available at https://publons.com/publon/10.1111/irv.12959.

## Supporting information


**Figure S1.** Map of the 17 cities in Shandong Province (red = coastal zone, blue = inland zone).
**Figure S2.** Longitude gradients in periodicity of the IVB epidemic.
(Panel A) Amplitude of the annual periodicity. (Panel B) Peaking time of the annual periodicity. Symbol size is proportional to the number of ILI cases in each city. Black solid lines represent linear regression fit (regression weighted by mean annual number of cases of ILI cases). *P* and R^2^ values are given on the graphs. Colors represent different city types (red = coastal zone, blue = inland zone). (Panel C) Amplitude of the annual cycle from pale red (low) to red (high). (Panel D) Peaking time of the influenza B epidemic, in weeks from Jan 1st. Timing is colour coded from pale green (low) to green (high).

## Data Availability

Research data are not shared.
