# Supplementary material for: Incidence, circulation, and spatiotemporal analysis of seasonal influenza in Shandong, China, 2008–2019: A retrospective study
Source: Influenza Other Respir Viruses. 2022 Jan 11;16(3):594–603. doi: 10.1111/irv.12959 (PMC8983897; doi:10.1111/irv.12959)
Supplement: Supplementary file 1 — Figure S1. Map of the 17 cities in Shandong Province (red = coastal zone, blue = inland zone). Figure S2. Longitude gradients in periodicity of the IVB epidemic. (Panel A) Amplitude of the annual periodicity. (Panel B) Peaking time of the annual periodicity. Symbol size is proportional to the number of ILI cases in each city. Black solid lines represent linear regression fit (regression weighted by mean annual number of cases of ILI cases). P and R2 values are given on the graphs. Colors represent different city types (red = coastal zone, blue = inland zone). (Panel C) Amplitude of the annual cycle from pale red (low) to red (high). (Panel D) Peaking time of the influenza B epidemic, in weeks from Jan 1st. Timing is colour coded from pale green (low) to green (high). [file IRV-16-594-s001.docx]

# Supplementary Information


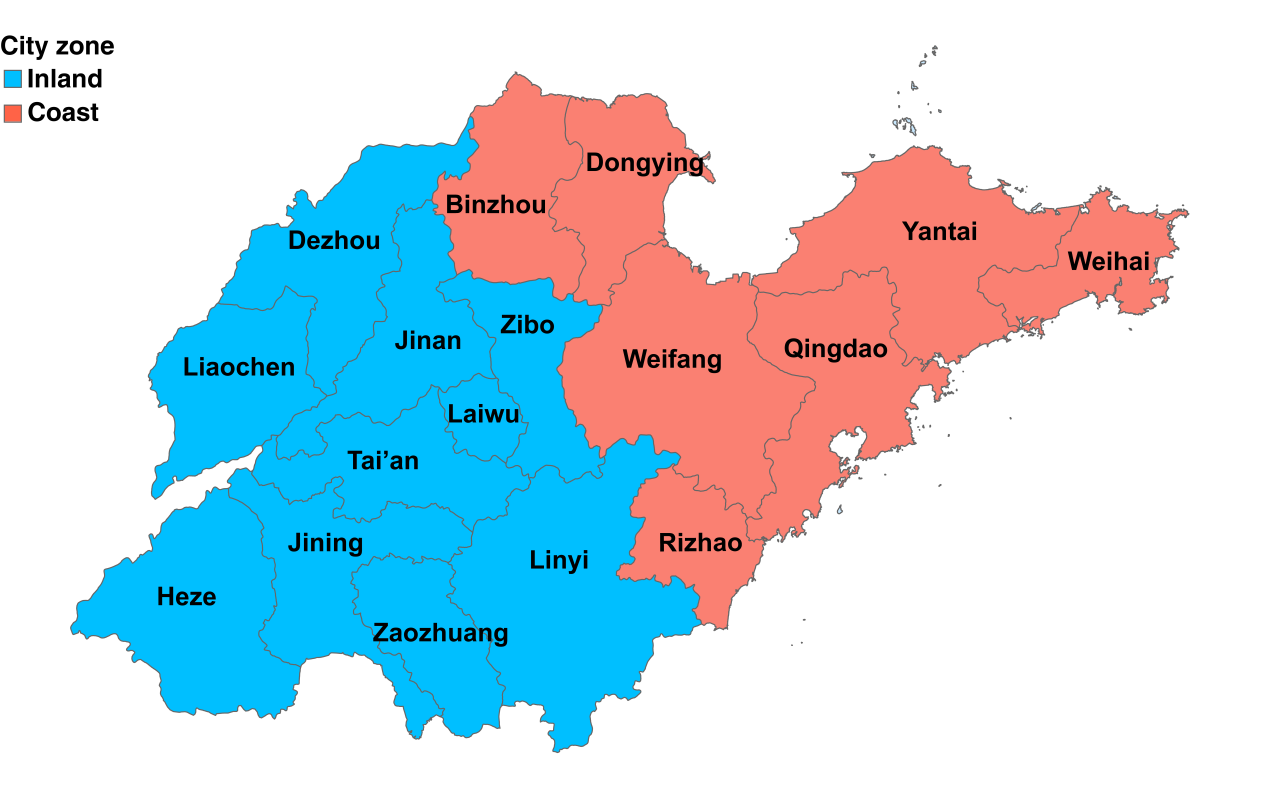


**Figure S1. Map of the 17 cities in Shandong Province (red = coastal zone, blue = inland zone).**


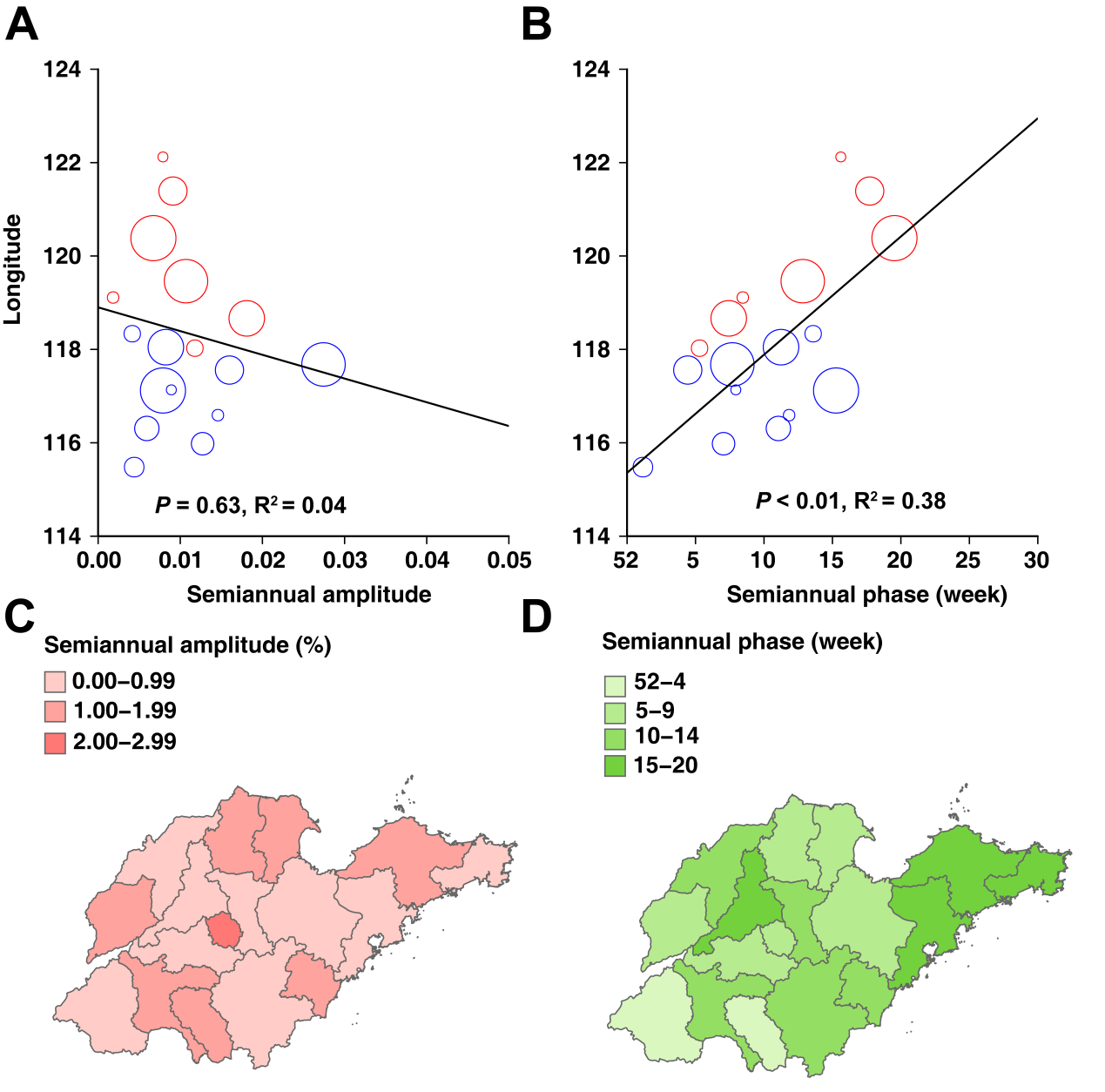


**Figure S2. Longitude gradients in periodicity of the IVB epidemic.**

(Panel A) Amplitude of the annual periodicity. (Panel B) Peaking time of the annual periodicity. Symbol size is proportional to the number of ILI cases in each city. Black solid lines represent linear regression fit (regression weighted by mean annual number of cases of ILI cases). *P* and R^2^ values are given on the graphs. Colors represent different city types (red = coastal zone, blue = inland zone). (Panel C) Amplitude of the annual cycle from pale red (low) to red (high). (Panel D) Peaking time of the influenza B epidemic, in weeks from Jan 1st. Timing is colour coded from pale green (low) to green (high).
